# Antibacterial potential of silver–selenium nanocomposites in mitigating fire blight disease in *Pyrus communis* L

**DOI:** 10.3389/fpls.2025.1541498

**Published:** 2025-03-12

**Authors:** Muhammad Imran, Muhammad Umer, Naveed Iqbal Raja, Fozia Abasi, Nimra Sardar, Ubaidur Rahman, Syed Azaz Mustafa Naqvi, Muhammad Yousuf Jat Baloch, Abdulwahed Fahad Alrefaei

**Affiliations:** ^1^ Department of Botany, PMAS Arid Agriculture University, Rawalpindi, Punjab, Pakistan; ^2^ School of Environmental Science and Engineering, Shandong University, Qingdao, China; ^3^ Department of Zoology, College of Science, King Saud University, Riyadh, Saudi Arabia

**Keywords:** *Pyrus communis* L. nanocomposite, fire blight, silver nanoparticles, selenium nanoparticles, *Erwinia amylovora*

## Abstract

*Pyrus communis* L. is a vital fruit tree known for its nutritional and economic importance. Thus, for humans, it is an essential element for their balanced nutritional diet, as it contains the major dietary fibers, vitamins, and minerals. All of these nutritionally important aspects decrease with the impact of disease fire blight. *Erwinia amylovora* is a causative agent of fire blight. This infection causes a considerable loss in the production of *Pyrus communis* L. Annually, approximately 50% of pear fruit in Pakistan is misplaced because of these illnesses. Therefore, we propose nanotechnology remediation to treat pear plants and obtain the desired yield. In this regard, an experiment was designed to treat infected plants with different concentrations of silver–selenium nanocomposites, which was based on a literature review that indicated the antimicrobial activities of silver and selenium nanoparticles. Silver–selenium nanocomposites were prepared using a green synthesis method, and their synthesis was confirmed using characterization techniques. The experiment was performed at a farmhouse in Chakwal district, Punjab, Pakistan. The experimental results showed increased morphological, physiological, and biochemical parameters. In this regard, the best treatment remained at 50 ppm for the Ag–Se nanocomposite, which improved the plant in different aspects. At the same time, they have improved fruit metrics, such as vitamin C, pH, and juice content. Thus, these results show a possible improvement in enhancing the resistance against fire blight by using green-synthesized Ag–Se NCs. Further studies are needed to understand fully the molecular mechanisms and actions of *Pyrus communis* L. in treating fire blight disease and to establish the optimal treatment plan.

## 1 Introduction

One of the most essential fruits cultivated in temperate regions is the pear, scientifically known as *Pyrus communis* L. They are a vital fruit crop not just in Europe but also in the temperate parts of the world as they have good economic status (Muller, 2024). Pear trees grow well in the temperate zone and are very responsive to technology applications, which may increase their yield and productivity. They are sweet and have a good smell. However, they are also a great source of Vitamin C and fiber. Furthermore, the low sodium content makes them a healthy intake source if consumed freshly. Pears are versatile and suitable adopters of conservation and transport; thus, this character makes them count in processed foods (Colavita et al., 2021). In Pakistan, according to the statistics given by the Food and Agriculture Organization (2022), the harvested area for the pear remains at 33,628 ha while the production of pear stands at 10,797.42 tons (https://www.fao.org/faostat/en/#data/QCL). The main cause of *Pyrus communis* yield loss is biotic stress. This biotic stress is caused by fire blight, the most serious disease that causes great yield loss worldwide.

The leading cause of the fire blight is *Erwinia amylovora*, which belongs to a gram-negative type of bacteria. This also disturbs the whole Rosaceae family of many species (Hafez et al., 2020). In California, USA, in 1976, the fire blight disease caused damage to many pear trees. The estimation of the loss was about 4.7 million dollars. In the 1990s, a significant fire blight infection caused damage of almost 3.8 million dollars in South Western Michigan. However, in 1998, a fire blight disease infected 80% of the cultivated area of pears in Egypt. Later, in the Northwest of America, the fire blight disease caused a significant loss of about 68 million dollars. In New Zealand, however, it has caused a loss of 10 million dollars. Meanwhile, in Italy and Lebanon, almost 5 million trees were damaged. In Morocco, in 2006, the fire blight disease spread to the other species of the family Rosaceae, which caused severe damage to the yield (Shoeib et al., 2017). The optimum temperature for the disease is about 18°C, while the warm season is much more encouraging for its growth and propagation. However, the functionality and appearance are improved with the increase in temperature (Santander and Biosca, 2017). This infection ultimately affects all the aerial structures of plants and makes them die. This pathogen is spread to the hypanthium from the stigma by the moisture of the surface and eventually impacts through the holes of nectar (Pusey et al., 2011). This disease needed to be settled effectively, requiring new studies and approaches.

The bacteria have developed resistance against the antibiotics, which were previously effective against them. Thus, nanotechnology emerged as a potential application in chemistry, pharmaceuticals, agriculture, and medicine (Colson et al., 2021; Elkady et al., 2024). The nanocomposites bring a novel approach that can increase flexibility and chemical and physical properties. These novel materials have the property of multifunctioning, which helps them to be versatile because they are the sum of multiple polymers (Al-Johani and Salam, 2011). It has been reported in several studies that the silver selenium nanocomposite has antimicrobial activities, specifically defined by the research done by (Ahmad et al., 2020). Thus, in this study, we aimed to apply silver–selenium nanocomposite to counter the disease of fire blight and provide an innovative and effective method for mitigating fire blight. As part of the research project, the following objectives were kept in mind:

1. Green synthesis and characterization of the plant-based  Ag–Se nanocomposite.

2. Assessment of morphological, physiological, and biochemical  attributes in response to the Ag–Se nanocomposite in *Pyrus  communis* against fire blight.

3. Assessment of fruit quality of *Pyrus communis* in response to  the Ag–Se nanocomposite.

## 2 Material and method

### 2.1 Plant material and experimental design

Healthy *Pyrus communis* L. plants of uniform size and age were selected for the study. These plants were grown in a farmhouse in District Chakwal, Punjab, Pakistan. The plants were distributed into four groups at random (**Table 1**). 

**Table 1:** Experimental layout for treatments on *Pyrus communis* L.

Sr. no.	Treatment group	Description
1	Control (normal)	No disease, no nanoparticles (NPs)
2	Control (−ve)	Disease only (no NPs applied)
3	Control (+ve, NM 25 ppm)	25 ppm nanoparticles only
4	Control (+ve, NM 50 ppm)	50 ppm nanoparticles only
5	Control (+ve, NM 75 ppm)	75 ppm nanoparticles only
6	Control (+ve, NM 100 ppm)	100 ppm nanoparticles only
7	Ag–Se nanocomposite (25 ppm) + disease	25 ppm Ag–Se nanocomposite + disease
8	Ag–Se nanocomposite (50 ppm) + disease	50 ppm Ag–Se nanocomposite + disease
9	Ag–Se nanocomposite (75 ppm) + disease	75 ppm Ag–Se nanocomposite + disease
10	Ag–Se nanocomposite (100 ppm) + disease	100 ppm Ag–Se nanocomposite + disease

### 2.2 Green synthesis and characterization of the Ag–Se nanocomposite

A green silver–selenium (Ag–Se) nanocomposite was synthesized by making plant extract using the method and precautions prescribed by Banerjee et al. (2014). However, the preparation of the silver–selenium nanocomposite was done by modifying the method described (Sibiya and Moloto, 2017). 5 mL of crude aqueous extract and 50 mL of 1 mM AgNO_3_ were mixed to begin the synthesis of the nanocomposite. This mixture was constantly heated at 50°C for about an hour, and subsequently, 5 mL of the aqueous extract was combined with 50 mL of 1 mM Na_2_SeO_3_, the selenium precursor. The reaction combination constantly swirled for 4 h at 80°C. After 15 min of centrifuging at 6,000 rpm, the excess fluid was disposed of. The pellet became reconstituted with the help of the 18-MW Milli-Q water. Later, different characterization techniques were used for the characterization purpose of the nanocomposite, including scanning electron microscopy (SEM), energy-dispersive X-ray (EDX), X-ray diffraction (XRD), and Fourier-transform infrared spectroscopy (FTIR). Dispersed individual solutions of silver and selenium nanoparticles were prepared, and equal amounts of both were added to a clean beaker. The beaker was put in the water bath ultrasonic processor, setting the ultrasonic frequency to 40 kHz and powering 100 W, and then the mixture was processed for 30 min to acquire homogeneity, proper dispersion, and the right interaction among the nanoparticles. The temperature was kept below 30°C by cooling the water bath with ice cubes while sonicating samples. It is recommended not to filter samples after sonication but instead to go for the next step of treatment like hydrothermal treatment or direct drying according to the type of application (Zhang et al., 2019).

### 2.3 Field experiment

The treatments were applied using foliar application at different concentrations on marked plants of *Pyrus communis* L. with three replicates. First, the morphological parameters were evaluated, which included the plant height, stem diameter, leaf area, disease severity index, and percentage of infected leaves.

### 2.4 Physiological parameters

The following parameters were selected for the physiological study.

#### 2.4.1 Membrane stability index

The membrane stability index was measured using the method described by Sairam (1994). The formula used


Membrane stability index = [1-C1/C2]×100

where C1 and C2 are electrical conductivities.

#### 2.4.2 Chlorophyll content

A leaf of about 20 g in weight was crushed in 80% of acetone. After crushing and filtration, the wavelengths were measured at 645, 652, and 653 (Arnon, 1949).

### 2.5 Biochemical parameters

The following biochemical parameters were evaluated for the biochemical studies.

#### 2.5.1 Phenolic and flavonoid contents

The phenolic content was quantified using the Singleton et al. (1999) method, while the total flavonoid content was measured using the procedure prescribed by Heimler et al. (2005). Quercetin was used as a reference.

#### 2.5.2 Soluble sugar and proline contents

The phenol-sulfuric acid technique was used to determine the amount of soluble carbohydrates. For this, 0.5 g of fresh leaf material was employed. These leaves were mixed with 10 mL of 80% ethanol. After that, the aggregate was cooked in a water bath at 80°C for 1 h. After this process, test tubes were full of 0.5 mL of the sample extract and 1 mL of an 18% phenol answer. The mixture was allowed to incubate at ambient temperature. The mixture was quickly stirred after an hour of incubation and the addition of 2.5 mL of H_2_SO₄. The absorbance of each pattern was then measured at a wavelength of 490 nm using a spectrophotometer. The method outlined by Bates et al. (1973) was used to measure the proline content.

#### 2.5.3 Antioxidant activities

##### 2.5.3.1 2,2-Diphenyl-1-picrylhydrazyl activity

The 2,2-diphenyl-1-picrylhydrazyl (DPPH) radical scavenging assay measures the antioxidant activity of a plant extract (Locatelli et al., 2009). 300 µL of 100 µM DPPH solution were put into 300 µL of a plant extract in liquid form (methanol was used in the case of the control). The mixture was shaken and left for 20 min in a dark room at room temperature (25°C), and then the absorbance was measured at 515 nm. Standard curves of ascorbic acid or quercetin were used to quantify antioxidant activity.


1% = [(AO-A/AO)]×100

##### 2.5.3.2 2,2′-Azino-bis (3-ethylbenzothiazoline-6-sulfonic acid) assay

This straightforward method was employed to determine the antioxidant potential of the plant extract. 1 mL of 10-fold diluted in ethanol solution of 2,2′-azino-bis (3-ethylbenzothiazoline-6-sulfonic acid) (ABTS) (Re et al., 1999) and 300 µL of extract at the time of assay were taken, vortexed, and incubated for 10 min in the dark; and decolorization of the blue/green reaction mixture was recorded against a blank containing solvent at 734 nm. The inhibition percentage was determined:


1% = [(AO-A/AO)]×100

A solution of Trolox (1,000 µM) was used as a standard, and a Trolox solution was used for the formation of a standard curve for further calculations.

##### 2.5.3.3 Estimation of catalase, peroxidase, and superoxide dismutase activities

The approach defined by Aebi (1983) was used to evaluate the catalase (CAT) activity. Meanwhile, the peroxidase (POD) was determined using the methodology of Onsa et al. (2004). However, the superoxide dismutase (SOD) was done by the method used by Kumar et al. (2012).

### 2.6 Fruit quality

The following parameters have been ascertained.

#### 2.6.1 Juice content

According to Lacey et al. (2009), the juice’s weight becomes measured and documented in grams. We calculated the percentage of juice contents using the following formula:

Fruit juice percentage = juice weight ÷ fruit weight × 100.

#### 2.6.2 pH

The pH of the fruit was recorded using the pH meter. The electrode was dipped into the juice and measurements were noted.

#### 2.6.3 Determination of ascorbic acid

Ascorbic acid was measured using the method outlined by Pascal (1985). A specified volume of standard ascorbic acid solution (0.01–0.1 mL) was combined with 0.2 mL of hydrochloric acid solution, mixed thoroughly, and then diluted to a total volume in several 10-mL calibrated flasks to prepare suitable working solutions (1–10 µg g⁻¹). The absorbances of these solutions were measured at 243 nm, using demineralized water as a reference. Clear samples were analyzed directly, while samples with high carbon dioxide content were degassed through vigorous shaking, and samples with significant solid content were centrifuged to remove suspended matter.

## 3 Results

### 3.1 Characterization of the Ag–Se nanocomposite

**Figure 1:**
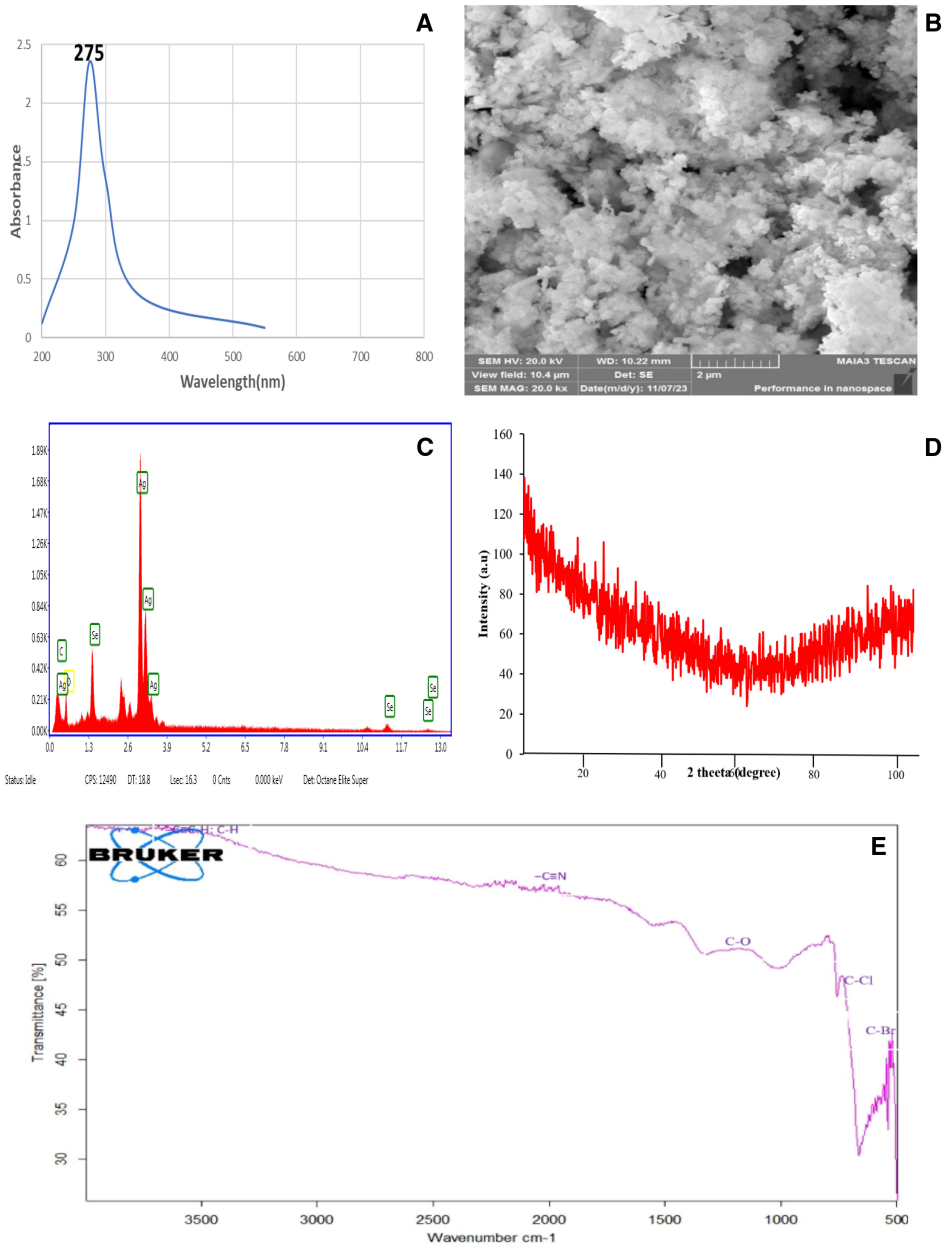
Comprehensive characterization of Ag–Se nanocomposites showcasing their structural, morphological, and functional properties: **(A)** UV-Vis spectroscopy analysis highlighting a strong absorbance peak at 275 nm, indicative of the optical properties of the Ag–Se nanocomposites. **(B)** Scanning electron microscopy (SEM) image revealed the surface morphology and nanoscale features of the Ag–Se nanocomposites, with agglomerated clusters and nanostructured textures. **(C)** Energy-dispersive X-ray (EDX) spectrum confirms the elemental composition of the nanocomposites, with distinct peaks corresponding to Ag and Se. **(D)** X-ray diffraction (XRD) analysis displays broad diffraction peaks, indicative of the amorphous nature of the nanoscale crystallinity of the Ag–Se nanocomposites. **(E)** Fourier transform infrared (FTIR) spectroscopy shows characteristic peaks corresponding to various functional groups (C–H, C=O, C–N, etc.) that may be involved in the stabilization and interaction of the nanocomposites.

The characterization of the Ag–Se nanocomposite by scanning electron microscopy confirmed that the nanoparticle size falls within the definition of nanoparticles. The EDX confirmed the purity level of the Ag–Se nanocomposite. UV-visible spectroscopy showed its peak at 275 nm (**Figure 1**). However, the characterization techniques FTIR and XRD have further confirmed the synthesis of the pure Ag–Se nanocomposite.

The XRD pattern of the Ag–Se nanocomposite shows broad and diffuse features without any sharp diffraction peaks, indicating a mostly amorphous structure or very small crystallite sizes. The absence of clear peaks suggests that the material does not have a well-defined crystalline order, which is common for amorphous materials or materials with nanoscale crystallites. This pattern suggests that the Ag–Se nanocomposite might have a high level of disorder, with the broad humps potentially representing short-range order within the nanocomposite.

The FTIR spectrum shows several distinctive absorption peaks that indicate the presence of specific functional groups in the sample. In particular, there are peaks at approximately 3,300 cm⁻¹, which could be attributed to O–H or N–H stretching vibrations. The peak around 2,200 cm⁻¹ suggests the presence of a nitrile group (C≡N). The absorption at around 1,700 cm⁻¹ indicates carbonyl (C=O) stretching, while the region near 1,200–1,000 cm⁻¹ corresponds to C–O stretching, suggesting the existence of ether or alcohol groups. Furthermore, peaks in the range of 700–500 cm⁻¹ suggest the presence of halogen compounds, specifically C–Cl and C–Br bonds. This spectrum provides valuable information about the different chemical bonds present in the sample, indicating a complex mixture of organic and possibly inorganic components.

### 3.2 Morphological parameters

**Figure 2:**
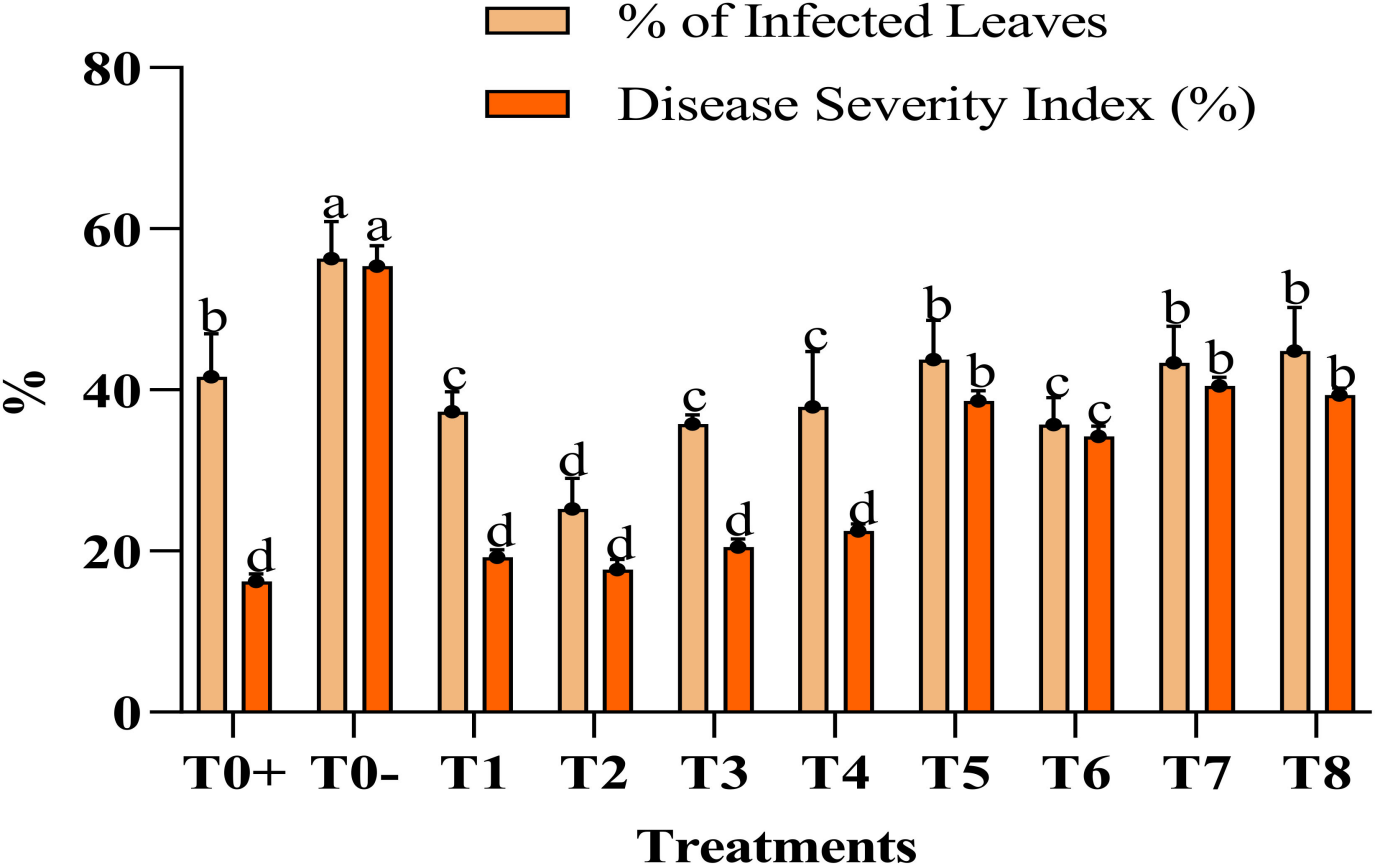
%age of infected leaves and disease severity index (%). Where T0+, control plants without any treatment; T0−, diseased plants without Ag–Se NC application; T1, plants treated with 25 ppm Ag–Se NCs; T2= plants treated with 50 ppm Ag–Se NCs; T3= plants treated with 75 ppm Ag–Se NCs; T4= plants treated with 100 ppm Ag–Se NCs; T5= plants treated with 25 ppm Ag–Se NCs+ *Erwinia amylovora*; T6= plants treated with 50 ppm Ag–Se NCs+ *Erwinia amylovora*; T7= plants treated with 75 ppm Ag–Se NCs+ *Erwinia amylovora*; T8= plants treated with 100 ppm Ag–Se NCs+ *Erwinia amylovora*.

The application of the Ag–Se nanocomposite led to a significant boost in the morphological characters. Specifically, foliar treatment with the Ag–Se nanocomposite has demonstrated greater efficacy in enhancing these parameters than untreated plants. The study examined plant height using different concentrations of the Ag–Se nanocomposite. Among the plants, the T2 plant treated with (50 ppm Ag–Se NCs) displayed the highest height, measuring 50.33 inches, whereas the T0 plant (untreated and diseased) exhibited the lowest height of 35.33 inches (**Table 2**). However, the other parameters showed the same pattern as the applied treatment of 50 ppm Ag–Se NCs, which increased the stem diameter and leaf area by 16.8% and 20%, respectively (**Figure 2**). The disease severity index and the percentage severity index revealed that the T2 treatment performed the best and reduced the rate of the disease severity index and percentage of infected leaves by 8% and 39%, respectively.

**Table 2:** Plant height, stem diameter, and leaf area are shown in mean ± standard deviation values.

Treatments	Plant height (inches) Mean ± S.D	Stem diameter (cm) Mean ± S.D	Leaf area (cm) Mean ± S.D
To+	43.333 ± 0.577	1.600 ± 0.050	13.977 ± 0.696
To-	35.333 ± 0.577 ^**^	1.200 ± 0.100 ^**^	8.242 ± 0.273 ^**^
T1	45.667 ± 0.577 ^**^	1.667 ± 0.208 ^**^	14.475 ± 0.582 ^**^
T2	50.333 ± 0.577 ^**^	1.947 ± 0.045 ^**^	16.792 ± 0.684 ^**^
T3	46.333 ± 0.577 ^**^	1.783 ± 0.076 ^**^	13.698 ± 0.702 ^**^
T4	46.000 ± 0.500 ^**^	1.700 ± 0.173 ^**^	14.197 ± 1.014 ^**^
T5	38.507 ± 0.491 ^**^	1.300 ± 0.050 ^**^	10.998 ± 0.640 ^**^
T6	40.877 ± 0.595 ^**^	1.517 ± 0.076 ^**^	11.970 ± 0.489 ^**^
T7	37.483 ± 0.475 ^**^	1.433 ± 0.076 ^**^	10.772 ± 0.381 ^**^
T8	36.000 ± 0.500 ^**^	1.450 ± 0.050 ^**^	11.777 ± 0.821 ^**^

Where T0+ = control plants without any treatment, T0−, diseased plants without Ag–Se NCs application; T1, plants treated with 25 ppm Ag–Se NCs; T2, plants treated with 50 ppm Ag–Se NCs; T3, plants treated with 75 ppm Ag–Se NCs; T4, plants treated with 100 ppm Ag–Se NCs; T5, plants treated with 25 ppm Ag–Se NCs+ *Erwinia amylovora*; T6, plants treated with 50 ppm Ag–Se NCs+ *Erwinia amylovora*; T7, plants treated with 75 ppm Ag–Se NCs+ *Erwinia amylovora*; T8, plants treated with 100 ppm Ag–Se NCs+ *Erwinia amylovora*. Values are written as mean ± standard deviation (S.D.); n, number of replicates (n=3). Significant differences **p < 0.01 compared with the Control group for Positive (T0+) and compared with the other treatment groups.

### 3.3 Physiological parameters

#### 3.3.1 Membrane stability index

**Figure 3:**
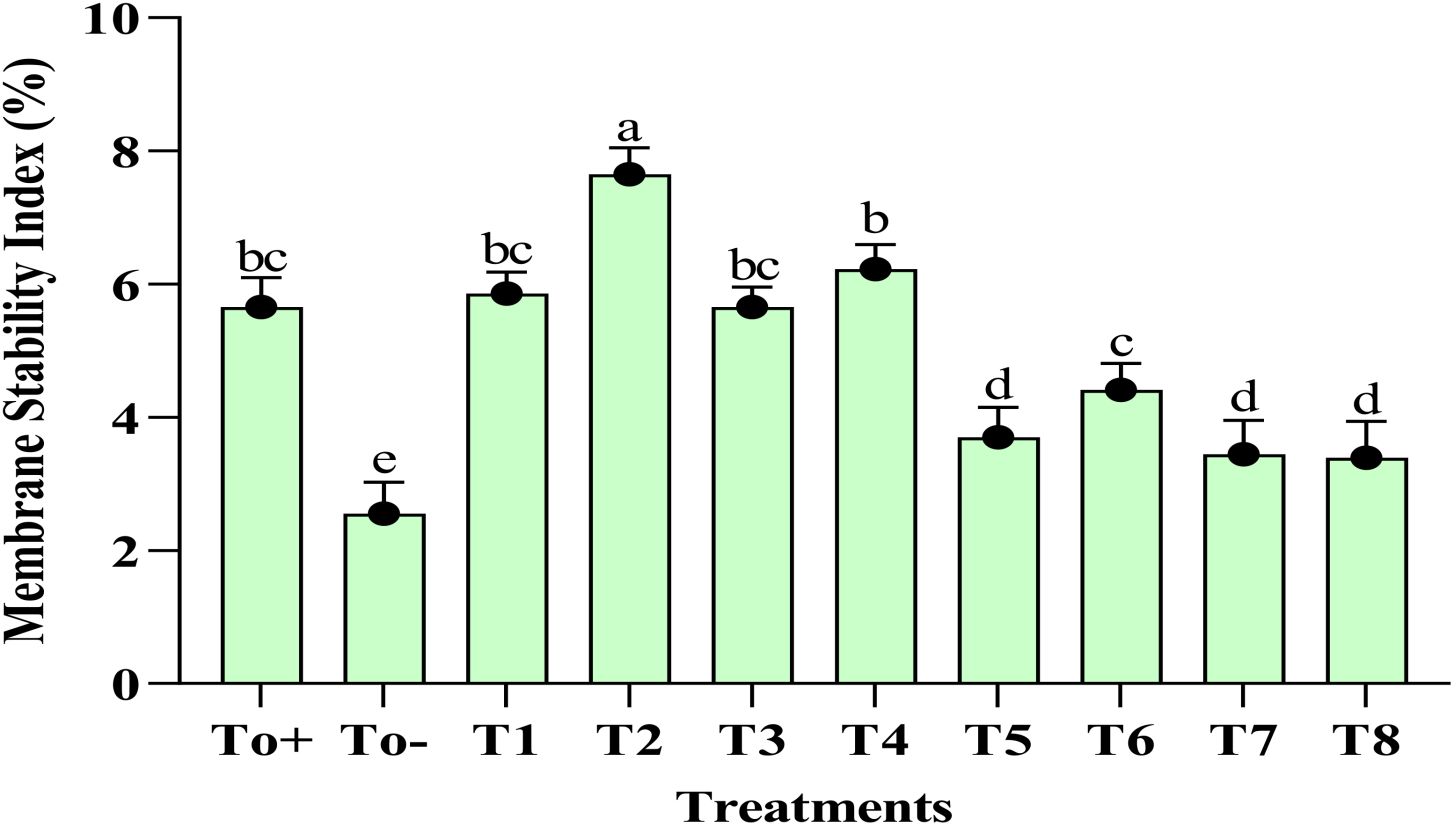
Membrane stability index. Where T0+, control plants without any treatment; T0−, diseased plants without Ag–Se NC application; T1, plants treated with 25 ppm Ag–Se NCs; T2= plants treated with 50 ppm Ag–Se NCs; T3= plants treated with 75 ppm Ag–Se NCs; T4= plants treated with 100 ppm Ag–Se NCs; T5= plants treated with 25 ppm Ag–Se NCs+ *Erwinia amylovora*; T6= plants treated with 50 ppm Ag–Se NCs+ *Erwinia amylovora*; T7= plants treated with 75 ppm Ag–Se NCs+ *Erwinia amylovora*; T8= plants treated with 100 ppm Ag-Se NCs+ *Erwinia amylovora*.

The plant treated with the 50-ppm Ag–Se NCs showed the best stability index of the membrane. The highest recorded membrane stability index % was at T2 (50 ppm Ag–Se NCs), which was 7.65%, while the lowest was observed at T0− (diseased only), 2.55%. **Figure 3** illustrates the varying MSI concentrations resulting from the foliar application of Ag–Se NCs. However, the lowest of the responses is shown by T_0_ negative. Meanwhile, the second-best treatment remains to be T4.

#### 3.3.2 Chlorophyll content

**Figure 4:**
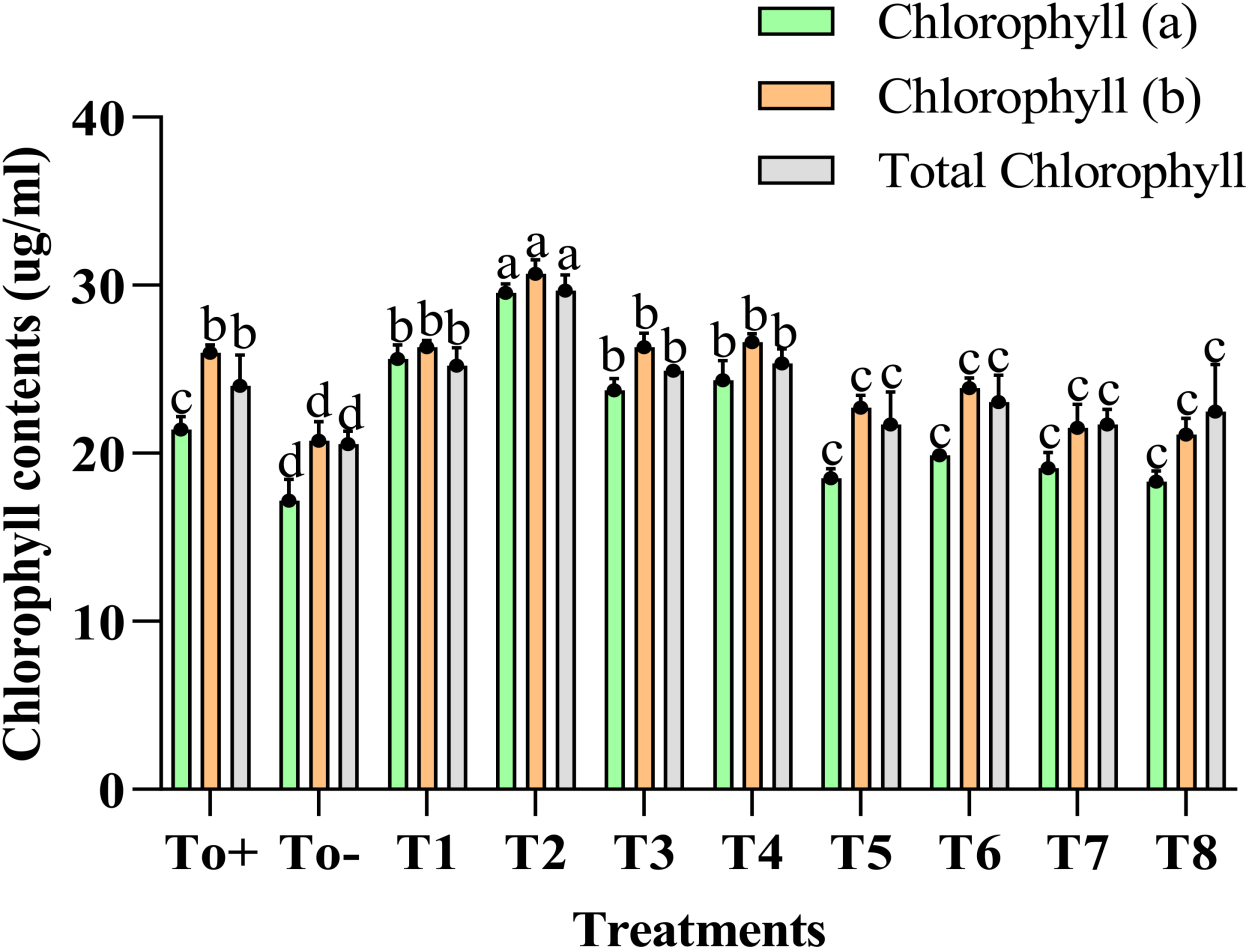
Chlorophyll contents. Where T0+, control plants without any treatment; T0−, diseased plants without Ag–Se NC application; T1, plants treated with 25 ppm Ag–Se NCs; T2= plants treated with 50 ppm Ag–Se NCs; T3= plants treated with 75 ppm Ag–Se NCs; T4= plants treated with 100 ppm Ag–Se NCs; T5= plants treated with 25 ppm Ag–Se NCs+ *Erwinia amylovora*; T6= plants treated with 50 ppm Ag–Se NCs+ *Erwinia amylovora*; T7= plants treated with 75 ppm Ag–Se NCs+ *Erwinia amylovora*; T8= plants treated with 100 ppm Ag-Se NCs+ *Erwinia amylovora*.

**Figure 4** explains the impact of different treatments on the chlorophyll content of the plant. The chlorophyll a, b and total chlorophyll enhanced to a great extent with the application of 50 ppm Ag–Se NCs. The increase in the chlorophyll A content compared with the negative control was 38%. Meanwhile, the increase in chlorophyll B was about 18%. However, the total chlorophyll content was believed to be increased by 23%. At the same time, the lowest of the results were evaluated in the To− (–ve).

### 3.4 Biochemical analysis

**Figure 5:**
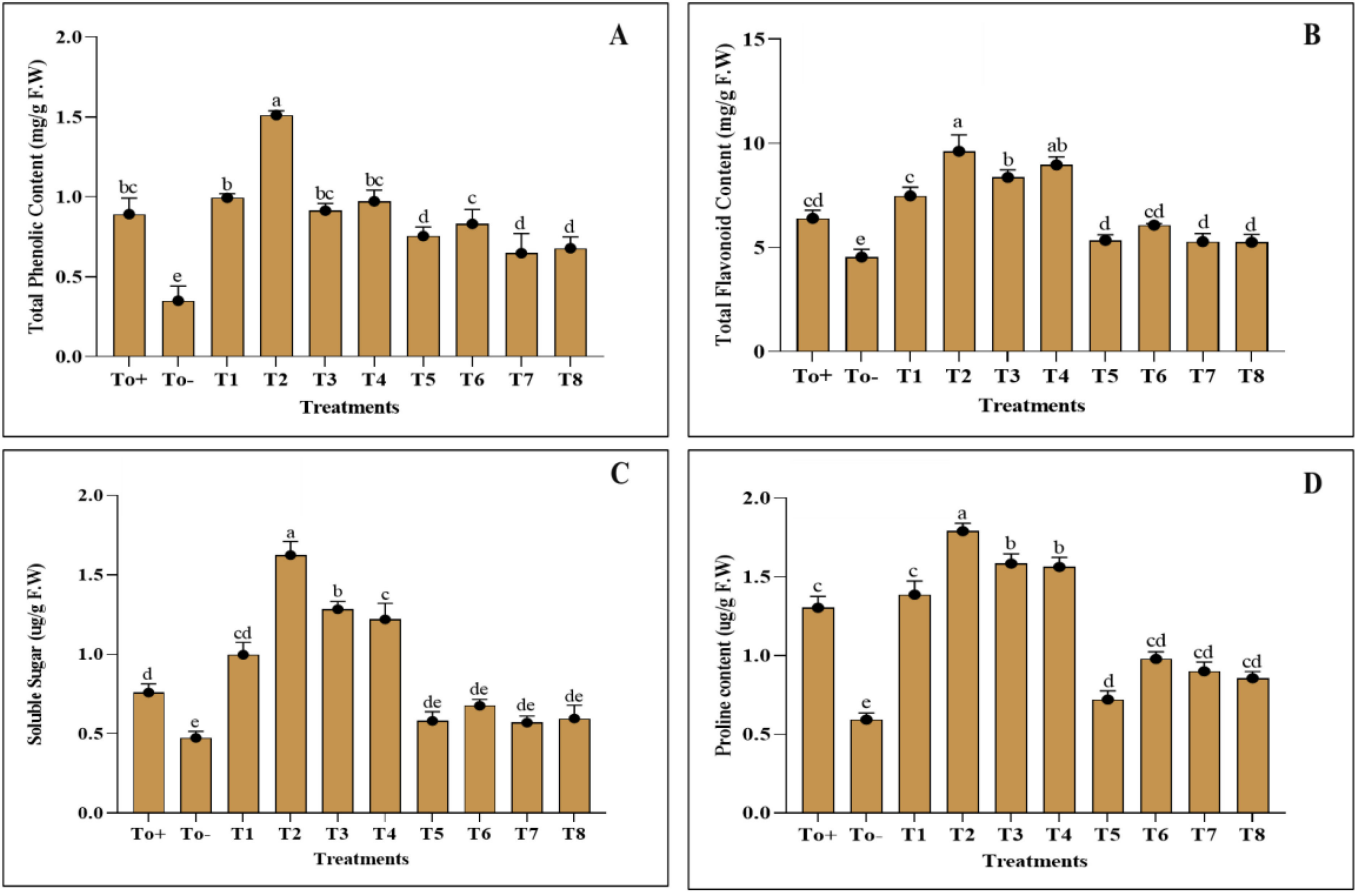
Biochemical activities. **(A)** Total phenolic content. **(B)** Total flavonoid content. **(C)** Soluble sugar. **(D)** Proline content. Where T0+, control plants without any treatment; T0−, diseased plants without Ag–Se NC application; T1, plants treated with 25 ppm Ag–Se NCs; T2= plants treated with 50 ppm Ag–Se NCs; T3= plants treated with 75 ppm Ag–Se NCs; T4= plants treated with 100 ppm Ag–Se NCs; T5= plants treated with 25 ppm Ag–Se NCs+ *Erwinia amylovora*; T6= plants treated with 50 ppm Ag–Se NCs+ *Erwinia amylovora*; T7= plants treated with 75 ppm Ag–Se NCs+ *Erwinia amylovora*; T8= plants treated with 100 ppm Ag-Se NCs+ *Erwinia amylovora*.

The application of the nanocomposite has impacted the biochemical attributes; however, the negative control T_0_− has adversely affected the biochemical parameters of plants. The phenolic and flavonoid content of the plant was best increased when 50ppm of nanocomposite was applied, and the increase in the phenolic and flavonoid contents was about 65% and 50%, respectively (**Figure 5**). The other evaluated parameters, soluble sugar and proline, have been enhanced best with 50 ppm, and the increase in the percentage as compared with the negative control was 114% and 37%. **Figure 5** further elaborates on the values of applied treatments.

### 3.5 Antioxidant activities

**Figure 6:**
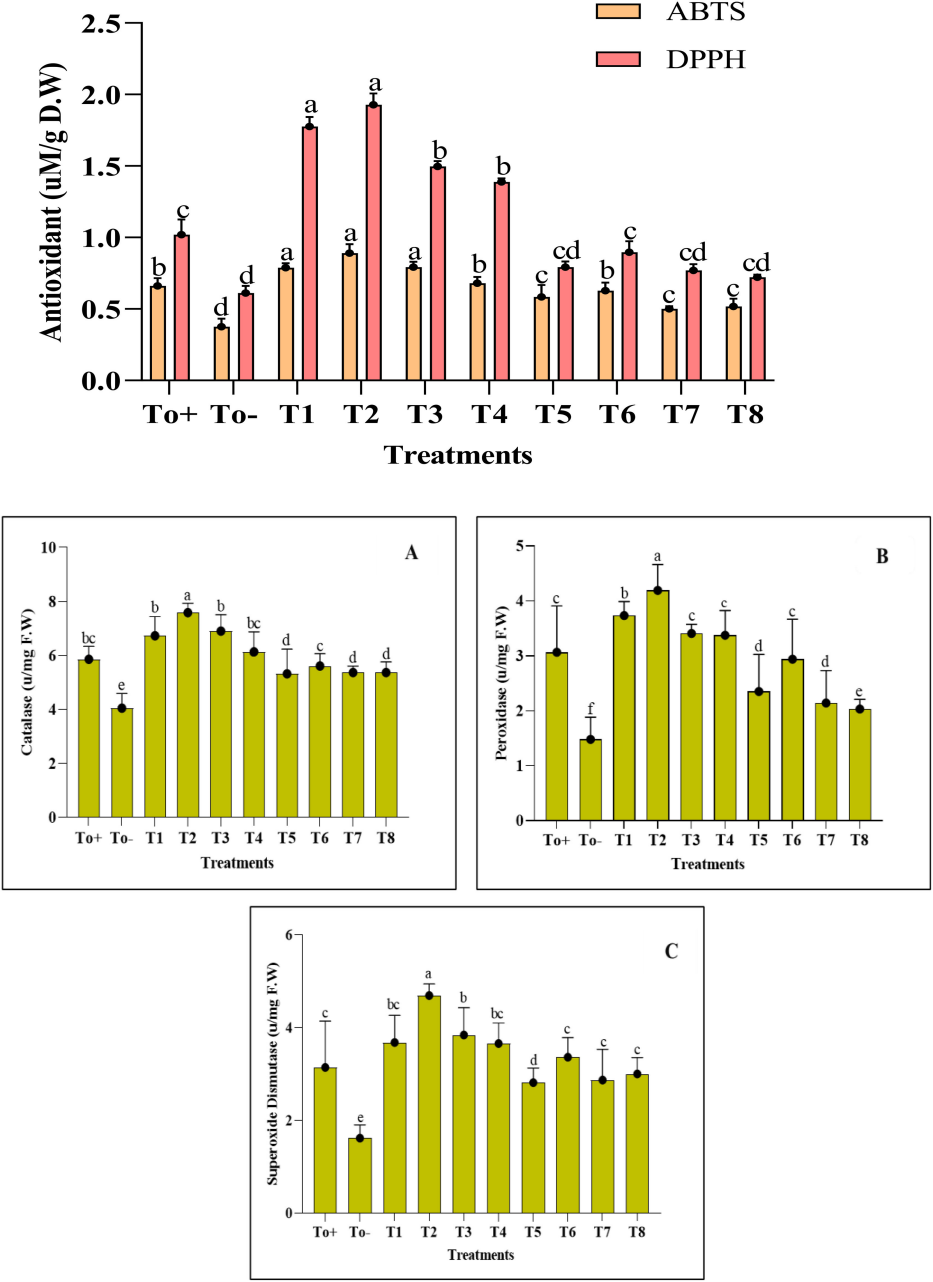
Antioxidant activity. **(A)** Catalase. **(B)** Peroxidase. **(C)** Superoxide dismutase. Where T0+, control plants without any treatment; T0−, diseased plants without Ag–Se NC application; T1, plants treated with 25 ppm Ag–Se NCs; T2= plants treated with 50 ppm Ag–Se NCs; T3= plants treated with 75 ppm Ag–Se NCs; T4= plants treated with 100 ppm Ag–Se NCs; T5= plants treated with 25 ppm Ag–Se NCs+ *Erwinia amylovora*; T6= plants treated with 50 ppm Ag–Se NCs+ *Erwinia amylovora*; T7= plants treated with 75 ppm Ag–Se NCs+ *Erwinia amylovora*; T8= plants treated with 100 ppm Ag-Se NCs+ *Erwinia amylovora*.

The activity of enzymatic and non-enzymatic antioxidants has shown an increase with the application of nanocomposite. Different ranges of treatments were evaluated, and the best of the treatments remained at 50 ppm for Ag–Se NCs. ABTS and DPPH showed an increase of 34% and 89% as compared with the negative control, while the CAT, POD, and SOD have shown an increment of about 29%, 36%, and 49%, respectively (**Figure 6**). Further details of the impact of different treatments applied and the control group provide confidence in the thoroughness of our research and the reliability of our results.

### 3.6 Fruit quality

#### 3.6.1 Juice content and pH

**Figure 7:**
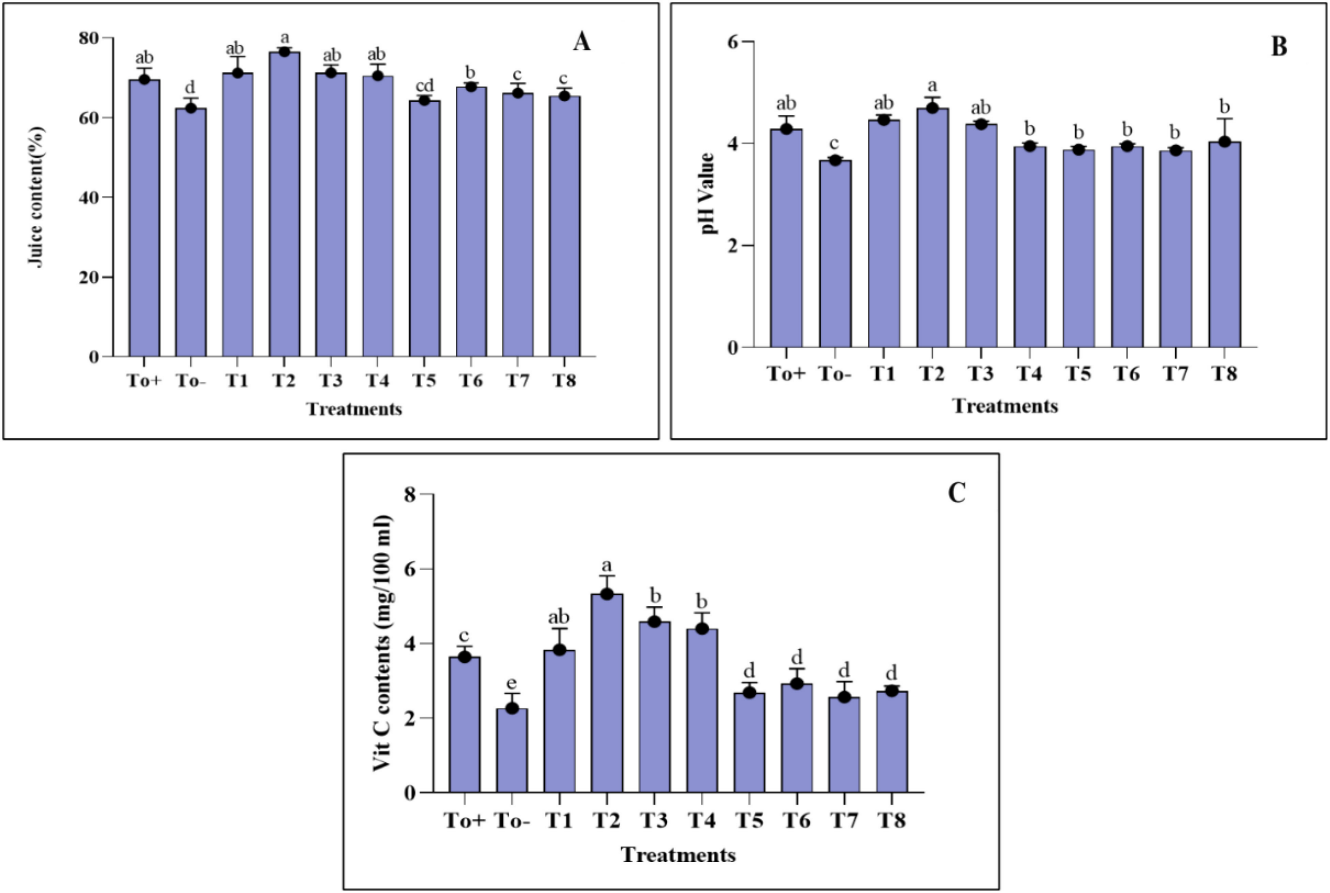
Fruit quality parameters. **(A)** Juice content. **(B)** pH value. **(C)** Vitamin C content. Where T0+, control plants without any treatment; T0−, diseased plants without Ag–Se NC application; T1, plants treated with 25 ppm Ag–Se NCs; T2= plants treated with 50 ppm Ag–Se NCs; T3= plants treated with 75 ppm Ag–Se NCs; T4= plants treated with 100 ppm Ag–Se NCs; T5= plants treated with 25 ppm Ag–Se NCs+ *Erwinia amylovora*; T6= plants treated with 50 ppm Ag–Se NCs+ *Erwinia amylovora*; T7= plants treated with 75 ppm Ag–Se NCs+ *Erwinia amylovora*; T8= plants treated with 100 ppm Ag-Se NCs+ *Erwinia amylovora*.

The juice content and pH of *Pyrus communis* were examined in response to different concentrations of plant-based synthesized Ag–Se NCs, as shown in (**Figure 7**). The pear plants infected with the pathogen only exhibited a decrease in juice content compared with the other treatments. The T2 plant (50 ppm Ag–Se NCs) had the highest juice content, measuring 76.58%. The lowest juice content was observed in the T0− plant (diseased only), at 62.39%. Meanwhile, the pH increase was observed best at T2 (50 ppm Ag–Se NCs), reaching a value of 4.7. The lowest pH was recorded at T0− (diseased only), with a value of 3.6. (**Figure 7**).

#### 3.6.2 Ascorbic acid determination

**Figure 7** demonstrates the investigation of the ascorbic acid content in *Pyrus communis*. It is evident that biotic stress negatively impacted pears’ ascorbic acid (AA) content. The highest amount of vitamin C was observed in T2 (50 ppm Ag–Se NCs), measuring 5.33 mg/100 mL. Conversely, the lowest vitamin C content was found in T0− (diseased only), measuring 2.27 mg/100 mL.

## 4 Discussion

Using modern technology that is environmentally friendly, cost-effective, and sustainable for managing plant disease is a highly viable option. This is particularly true due to the active application of nanotechnology in disease control. The nanoparticles are considered to be massive game changers in today’s world; however, their role can be regarded as more positive as compared with negative. They have the potential to improve agriculture and help farmers achieve sustainable growth (Rasouli et al., 2020). Nanoparticles, such as silver and selenium, synthesized through environmentally friendly methods, have been widely used to combat various plant pathogens. Producing the nanoparticles through the plant extract is an easy, rapid, and environmentally friendly technique (Nayeri et al., 2020). The unique qualities of green synthesized nanoparticles have led to a rise in demand for them. The silver nanoparticles have the potential against the microbes and can enhance the antioxidant activities (Jakinala et al., 2021). A study done by Alsulami et al. (2023) shows the antimicrobial potential of silver nanoparticles against the four harmful pathogens. However, the selenium nanoparticles also possess a great extent of antimicrobial activity that was shown in a study on wheat where it enhanced the resistance against the spot blotch bacterial disease and improved the wheat crop (Shahbaz et al., 2023a). The objective of the present investigation was to assess the antibacterial capabilities of selenium and silver nanoparticles against *Erwinia amylovora*, which causes fire blight in *Pyrus communis* L. According to Haleemkhan et al. (2015), various phytochemicals in medicinal flowers, flavonoids, phenols, and alkaloids create nanoparticles (NPs). It takes these secondary metabolites to transform precursor molecules into NPs. The chemical is a catalyst throughout the synthesis technique, while the discount approach is used. The steadiness of the resultant nanoparticles is also enhanced via the reducing agent in this process. Time, pH, and plant extract concentration are some variables that affect the production of selenium nanoparticles (Menon et al., 2020). The application of the nanocomposite was tested on *Pyrus communis* L. In terms of morphological characters, the nanocomposite has improved the pear’s plant height, leaf area, and stem diameter. Our results aligned with the previous research done by Eldin (2015). In this investigation, we have come across a fall in the chlorophyll content, and this decrease in the content is referred to as the impact of fire blight disease. Oxidative stress is the primary cause of a fall in the chlorophyll content and damage to its structure; this was previously observed in the studies done by Shahbaz et al. (2023b) and Wu et al. (2015). By applying nanocomposite in our research, we have seen that with the low concentration of the silver–selenium nanocomposite, the increase in chlorophyll a, b and total chlorophyll was found, increasing the photosynthesis rate. This is so because plants’ productivity increased after applying the silver–selenium nanocomposite. This may be referred to as the improvement in the growth parameters of the root, which leads to better absorption of the mineral nutrients that eventually increase the rate of photosynthesis and the growth parameters of the plant. It is now well documented that the lower concentration application of silver–selenium is essential for the increment in the stomatal conductance and the activity of RUBISCO that, in turn, increases the photosynthetic activity of photosystem II; this increase leads to enhanced plant productivity and rate of photosynthesis (El-Ramady et al., 2016; Jiang et al., 2015; Murali et al., 2023). A significant fall is seen in the membrane’s stability index due to the impact of the fire blight. However, the treated plant with 50 mg Ag–Se increased the stability index because AgNPs can elevate antioxidant levels, protecting cell membranes from oxidative stress. For instance, AgNPs derived from *Cordia myxa* exhibited high stability and antioxidant properties, potentially contributing to membrane integrity over time (Samari et al., 2019). The biotic stress also brings oxidative stress to the plant and makes it vulnerable. Therefore, applying nanoparticles may remain a beneficial treatment of the plant. The nanoparticles can enhance the secondary metabolism of plants by interfering with multiple signaling pathways. The first response of plants to the application of nanoparticles is the increased level of Ca_2+_, increment in the level of ROS, and the activation of the mitogen-activated protein kinase cascades. A link exists between ROS and the secondary response messengers that lead to secondary metabolism in transcriptional regulation. A link also exists among the ROS, the secondary signaling molecule, and the reactive oxygen species in the process of transcriptional regulation of the plants’ secondary metabolisms (Samynathan et al., 2023). The application of the silver–selenium nanocomposite has increased the plant’s antioxidant content. The biotic stress lowered the antioxidant system of the plant, as plants’ significant dependence is on the immune system for their protection against diseases. Therefore, the fire blight has damaged the antioxidant defense system to a great extent. However, as per the findings of this study, we have evaluated that the application of the nanocomposite increased the antioxidant elements, which include the POD, CAT, SOD, DPPH, and ABTS. This present study’s findings align with those of previous studies. The study reported that the application of selenium nanoparticles increased the SOD activity in the Sorghum plant facing stress conditions, which then limited the stress condition for the plant (Djanaguiraman et al., 2018). The study by Tasnim et al. (2024) showed the potential role of AgNPs in enhancing the ABTS and DPPS activities; this result also aligns with our current study. However, other investigations do support the increase in the antioxidant activity of plants, as indicated by the research done by Zahedi et al. (2019). Similarly, another study done by Jiang et al. (2017) indicated an increase in the SOD content in corn plants after the application of selenium nanoparticles in corn plants, which resulted in enhancing tolerance against stress in plants. The results of our antioxidant activity are in line with the findings done by Hussein et al. (2019). Applying the nanocomposite increased the plant’s total phenolic and flavonoid content. This phenomenon is because silver nanoparticles are best at upregulating gene expression, which induces the biosynthesis process of phenolic compounds (Xing et al., 2015). The flavonoid content that accumulates in the vacuole of epidermal layer cells in leaves is then helpful in lowering oxidative stress. Flavonoids possess the property of stopping hydrolytic and oxidative enzymes, ROS activity, and the anti-inflammation activity of plants (Atanassova et al., 2011). Our study has shown that applying the silver–selenium nanocomposite has increased plants’ phenolic and flavonoid content. The same result was shown in the study by Krishnaraj et al. (2012) and Bagherzadeh Homaee and Ehsanpour (2015). Fruit quality is important in fruit longevity and human nutrition (Neysanian et al., 2020). In our study, applying the nanocomposite improved the fruit quality parameters. The application of silver nanoparticles improved the quality of fruit as it contains imposing antifungal and antibacterial properties; this property helps in improving the fruit quality by stopping the pathogen growths such as Listeria monocytogenes and *Staphylococcus aureus* (Alharbi et al., 2024; Shahat et al., 2020).

## 5 Conclusion

This study evaluated the effectiveness of the silver–selenium nanocomposite against the fire blight on *Pyrus communis* L. The outcomes of this study revealed that the application of the silver–selenium nanocomposite improved plants’ morphological, physiological, biochemical, antioxidant, and juice contents. The best treatment remained to be T2, which has the most positive impact on the plant and increases the internal defense mechanism by enhancing the antioxidant activities. Silver and selenium nanoparticles possess antimicrobial activity that has lowered the effect of disease on plants and, therefore, helped mitigate the plant against biotic stress. Consequently, using the silver–selenium nanocomposite can be an effective treatment against the harmful disease of fire blight and can help enhance the yield of plants.

## Data Availability

The original contributions presented in the study are included in the article/supplementary material. Further inquiries can be directed to the corresponding authors.
